# Current status and variables associated with readiness for hospital discharge in patients with brucellosis in Ningxia, China: a cross-sectional study

**DOI:** 10.3389/fmed.2026.1795489

**Published:** 2026-08-12

**Authors:** Chune Gu, Yanling Si, Huijuan Wang

**Affiliations:** The Fourth People’s Hospital of Ningxia Hui Autonomous Region, Yinchuan, China

**Keywords:** brucellosis, discharge teaching quality, perceived social support, psychological distress, random forest, readiness for hospital discharge

## Abstract

**Objective:**

To explore the relevant influencing variables of discharge readiness in patients with brucellosis, so as to provide evidence-based references for implementing precise clinical intervention, optimizing discharge management and enhancing patients’ overall discharge readiness.

**Methods:**

Following the STROBE guidelines, a cross-sectional study was conducted. A total of 270 inpatients with brucellosis were enrolled via convenience sampling from September 2024 to July 2025 in the Department of Infectious Diseases of a tertiary specialized hospital in Ningxia. Data were collected on the day of hospital discharge using a general information questionnaire, the Readiness for Hospital Discharge Scale (RHDS), the Kessler Psychological Distress Scale (K10), the Multidimensional Scale of Perceived Social Support (MSPSS), and the Quality of Discharge Teaching Scale (QDTS). The variable selection and model construction process is as follows: First, using R Studio software, variables showing statistically significant differences in univariate and correlation analyses were included in the random forest model. The IncMSE score was used to assess the weight of each variable’s influence on discharge readiness; Next, Lasso analysis was applied to determine the optimal lambda value for selecting core independent variables. Finally, multiple linear regression analysis was performed using the Enter method (*α*_entry_ = 0.05, *α*_exit_ = 0.10) to identify the variables influencing discharge readiness.

**Results:**

The score of discharge readiness in brucellosis patients was 88.00 (73.00, 100.25), indicating a moderate level. The scores of the other scales were as follows: K10 24.00 (18.00, 29.00), MSPSS 58.00 (48.75, 66.25), and QDTS 175.00 (150.00, 199.00). Spearman correlation analysis showed that discharge readiness was negatively correlated with psychological distress (*r* = −0.249, *p* < 0.001). It was also positively correlated with perceived social support (*r* = 0.271, *p* < 0.001) and discharge teaching quality (*r* = 0.413, *p* < 0.001). Univariate analysis revealed statistically significant differences (*p* < 0.05) in discharge readiness scores among patients with brucellosis based on age, educational level, occupations, household income, marital status, medical expense payment methods, residency, and the presence of symptoms. The ranking of feature importance in the Random Forest model (sorted by IncMSE score from highest to lowest) was as follows: QDTS, MSPSS, educational level, K10, marital status, household income, age, occupation, medical expense payment methods, residency, and presence of symptoms. In the Lasso analysis, when lambda.1se = 4.02848, the optimal number of variables was 4. Combining this with the variable importance ranking from the random forest analysis, the top 4 variables were included in the multiple linear regression analysis. Residual tests indicated that the data exhibited an approximately symmetrical distribution around the origin, supporting the suitability of the linear regression model. The results showed that educational level, K10, MSPSS, and QDTS were independent predictors of discharge readiness (all *p* < 0.05), collectively explaining 28.4% of the total variance.

**Conclusion:**

Brucellosis patients had a moderate level of discharge readiness, Better discharge teaching quality, greater perceived social support, higher educational level, and lower psychological distress were associated with higher discharge readiness.

**Limitation:**

One limitation of this study is that the dataset was not split into training and validation sets for data analysis; therefore, the results are only of descriptive statistical value with limited generalizability.

## 1 Introduction

Brucellosis, also known as Malta fever or undulant fever, is a zoonotic infectious disease. It is induced by facultatively intracellular, Gram-negative, non-motile bacilli belonging to the genus *Brucella*, primarily the pathogenic species *Brucella melitensis*, *Brucella abortus* and *Brucella suis*, alongside several minor species, which invade hosts and lead to multi-organ systemic injury (1). *Brucella* invade the human body through the mucous membranes, broken skin, and respiratory tract, followed by transportation to regional lymph nodes via phagocytes (2). These bacteria establish long-term intracellular colonization by inhibiting macrophage apoptosis, subsequently disseminating to multiple reticuloendothelial organs through the bloodstream (2). This pathological process triggers acute bacteremia and the formation of typical granulomatous lesions (2). The predominant clinical manifestations of acute brucellosis consist of fever, profound fatigue, anorexia, weight loss, as well as myalgia and arthralgia. Given the non-specificity of these clinical symptoms, brucellosis is commonly misdiagnosed as malaria, typhoid fever, tuberculosis, lymphoma, and connective tissue diseases (2).

Globally, approximately 1.6 to 2.1 million new human cases are reported each year (2). China bears one of the highest brucellosis disease burdens in East Asia, with a rising incidence and expanding endemic areas; the incidence increased from 0.028 per 100,000 in 1993 to 4.69 per 100,000 in 2022 (3). Ningxia, together with its adjacent provinces including Inner Mongolia and Xinjiang, is part of a high-prevalence region for brucellosis in northern China. In the early 1990s, alongside socioeconomic development and the large-scale expansion of mutton sheep breeding in this region, local brucellosis outbreaks re-emerged and have exhibited a sustained and slow annual increasing trend. The monthly number of reported cases in the region rose gradually from less than 10 in 2004 to 200–300 in 2019 (4). A sharp surge in case counts was observed between 2020 and 2022, with the monthly peak reaching 827 cases in June 2022. Despite a slight epidemic decline during 2023–2024, the regional monthly average number of confirmed cases remained above 300 (4). Overall, the epidemiological burden of brucellosis in this region remains substantial, posing persistent challenges to brucellosis prevention and control. This disease not only seriously affects the physical and mental health of local residents, reduces their quality of life and working ability, but also impedes the development of local animal husbandry and socioeconomic progress, since it easily leads to livestock abortion and production reduction and restricts industrial scale development (5).

Brucellosis primarily affects middle-aged men and young adults, as well as farmers and herdsmen (3). If medication is not taken as prescribed, it can easily lead to chronic complications such as osteoarthritis, cardiovascular disease, and neurological disorders (6). Standardized clinical pathways, early targeted interventions, optimized diagnostic and therapeutic regimens, and the implementation of the tiered healthcare system have collectively contributed to an increase in hospital bed turnover rates and a reduction in length of stay (7). As a result, brucellosis patients often complete their post-acute symptom management and rehabilitation at home, presenting new challenges to the safety and continuity of care after discharge. Studies indicate that approximately 5–30% of brucellosis cases experience recurrence and readmission due to inappropriate antimicrobial selection and insufficient treatment duration (8). Therefore, evaluation of discharge readiness among this population is essential.

Discharge readiness is defined as a multidimensional construct that systematically assesses patients’ physiological stability, psychological adaptation, and social support resources to quantify their capacity for safe community reintegration and post-discharge recovery (9). Accumulating clinical evidence has confirmed that adequate discharge readiness is negatively correlated with the risks of post-discharge complications, unplanned readmission and all-cause mortality, namely, higher discharge readiness contributes to lower occurrence of these adverse clinical outcomes (10, 11). Psychological distress refers to a constellation of non-specific negative psychological states, including frustration, anxiety, and depression, experienced by individuals under stress (12). Among patients with brucellosis, the prevalence of anxiety and frustration is as high as 73.9% (13), which not only impairs their discharge readiness but also compromises their health-related quality of life (14). Discharge teaching and social support, as established predictors of discharge readiness, have been widely documented to be positively associated with higher levels of discharge readiness across patient populations (15, 16).

While existing research has explored discharge readiness among patients with infectious diseases such as tuberculosis and HIV/AIDS, there remains a gap in the literature regarding discharge readiness and its influencing variables among patients with brucellosis. In this study, variables refer to all measurable indicators associated with patients’ discharge readiness. Furthermore, previous studies have largely relied on linear regression models to identify influencing variables. These models struggle to address multicollinearity among multidimensional variables and fail to accurately quantify the true weight of each variable. Consequently, this study focuses on hospitalized patients with brucellosis and employs a composite analytical method combining the random forest model with Lasso regression. Specifically, the random forest model can quantitatively rank variables by importance and clarify the relative influence of each variable; Lasso regression, leveraging L1 regularization constraints, effectively eliminates multicollinearity interference and removes redundant variables, thereby achieving precise selection of core variables and variable dimensionality reduction. Based on existing theoretical and clinical research, this study proposes the following hypotheses: (1) Multidimensional variables such as psychological distress, social support, and health education quality are the core variables influencing the discharge readiness of patients with brucellosis; (2) The random forest-LASSO regression strategy systematically differentiates the effect magnitudes of multidimensional predictors via variable importance ranking and penalized dimensionality reduction, efficiently screening variables for priority intervention.

This study aims to identify the key variables influencing discharge readiness in patients with brucellosis. The findings will provide reliable empirical evidence for clinicians to develop individualized and standardized discharge intervention plans and offer significant clinical guidance for enhancing patients’ discharge readiness, improving post-discharge rehabilitation outcomes, and optimizing long-term prognosis.

## 2 Methods

### 2.1 Study design and participants

This study adopted convenience sampling for participant recruitment (17). Participants were consecutive inpatients diagnosed with brucellosis from the Department of Infectious Diseases at a tertiary specialty hospital in Ningxia. The enrollment period spanned from September 2024 to July 2025. All enrolled patients met the standardized inclusion and exclusion criteria and provided voluntary participation in this study. This sampling approach is suitable for observational studies targeting specific clinical diseases with limited case resources, enabling efficient sample collection under real clinical scenarios. Additionally, unified enrollment criteria were strictly implemented throughout the study to minimize potential selection bias. Inclusion criteria were as follows: (1) Patients with a first-time diagnosis of brucellosis based on the Expert Consensus on the Diagnosis and Treatment of Brucellosis; (2) Age ≥ 18 years; (3) Provided written informed consent and voluntarily participated in the study. Exclusion criteria were as follows: (1) Patients with other infectious diseases (e.g., AIDS, hepatitis B), severe organ failure (heart, lung, brain, liver, kidney, etc.), hemoptysis, or critical illness; (2) Pregnant women, patients with recurrent brucellosis, or patients with drug-resistant bacterial infections.

### 2.2 Sample size calculation

This single-center cross-sectional study calculated the sample size to ensure reliable estimation of discharge readiness scores. The following formula for multivariate analysis was used (18), *N* = (*UαS/δ*)^2^, where *α* represents the significance level, and *Uα* is the corresponding standard normal quantile. *S* stands for the standard deviation of discharge readiness scores. *δ* (delta) is the maximum allowed absolute error, namely the maximum acceptable difference between the sample mean and the true population mean of discharge readiness scores. A smaller *δ* value corresponds to higher estimation precision. With *α* = 0.05, the corresponding *Uα* was 1.96. The standard deviation of discharge readiness scores among patients with brucellosis was 3.0, as determined by the pilot study. Substituting these values with *δ* = 0.38 yielded a minimum sample size of 240. After accounting for a 10% proportion of invalid questionnaires, the required sample size was 264. Ultimately, 270 valid participants were enrolled, and the sample size met the requirements for the subsequent exploratory multivariate analysis.

### 2.3 Ethical considerations

This study involved human participants and was approval by the Ethics Committee of Ningxia Fourth People’s Hospital (Ethics Approval No: 202201). All participants provided written informed consent prior to enrollment. Confidentiality, anonymity, and data protection were maintained throughout the study.

### 2.4 Research tools

#### 2.4.1 General information questionnaire

A socio-demographic and clinical questionnaire was developed based on a literature review and group discussion by the research team. It included items such as gender, age, educational level, occupation, monthly per capita household income, marital status, medical payment method, place of residence, and presence of symptoms.

#### 2.4.2 Readiness for hospital discharge scale (RHDS)

The Readiness for Hospital Discharge Scale (RHDS) was originally developed by Weiss et al. (19) and subsequently translated and validated into Chinese by Lin et al. (20). It is designed to assess readiness for hospital discharge across diverse patient populations, encompassing three dimensions: personal status, coping ability, and expected support, with a total of 12 items. The total scale score is calculated as the sum of scores for all individual items. The scale uses a 0–10 numerical rating scale, where 0 indicates “not at all ready” and 10 represents “completely ready.” The Cronbach’s *α* coefficient for the scale was 0.89. Cronbach’s α coefficient is a statistical indicator used to evaluate the internal reliability of scales and is widely adopted for questionnaire reliability testing, with a value ranging from 0 to 1. a Cronbach’s *α* coefficient ≥ 0.8 indicates good scale reliability, 0.7 ≤ α< 0.8 represents acceptable reliability, and α < 0.7 suggests poor internal consistency reliability (21).

#### 2.4.3 Psychological distress scale (K10)

The Psychological Distress Scale (K10) was developed by Kessler and Mroczek (22) and first applied in China by Xu et al. (23). It is used to assess the frequency of non-specific psychological distress symptoms (e.g., depression, anxiety) experienced by individuals over the past 4 weeks. The scale employs a 5-point Likert scale, with scores ranging from 1 to 5 representing “none of the time,” “a little of the time,” “some of the time,” “most of the time,” and “all of the time,” respectively. It consists of 10 items, and the total score is calculated as the sum of scores for all individual items, with a total score range of 10–50. The Cronbach’s *α* coefficient for the scale was 0.89. Widely used in various populations both domestically and internationally, the K10 is a reliable tool for assessing population mental health status.

#### 2.4.4 Multidimensional scale of perceived social support (MSPSS)

The Multidimensional Scale of Perceived Social Support (MSPSS) was developed by Zimet et al. (24) and subsequently translated and validated into Chinese by Jiang (25). It contains 12 items and comprises three dimensions: family support, friend support, and significant other support. Each item is rated on a 7-point Likert scale ranging from 1 (strongly disagree) to 7 (strongly agree), with total scores ranging from 12 to 84. The scale is primarily used to assess an individual’s level of perceived social support, with higher scores indicating better perceived social support. The Cronbach’s *α* coefficient for the total scale was 0.88.

#### 2.4.5 Quality of discharge teaching scale (QDTS)

The Quality of Discharge Teaching Scale (QDTS) was developed by Weiss et al. (26). The Chinese version translated and validated by Wang et al. (27) was used in this study. The scale consists of 24 items across three dimensions: knowledge needed before discharge, knowledge actually received before discharge, and health teaching effectiveness. It uses a 0–10 numerical rating scale, with the total score calculated as the sum of the three dimension scores. Higher scores indicate better quality of discharge teaching. The Cronbach’s *α* coefficient for the scale was 0.92.

### 2.5 Data collection and quality control methods

On the day of discharge, after routine discharge procedures and health education were completed, paper questionnaires were distributed to patients meeting the inclusion and exclusion criteria. This timing ensured that the Readiness for Hospital Discharge Scale and the Quality of Discharge Teaching Scale were assessed in a manner consistent with their intended constructs. All questionnaires were checked on site for completeness and logical consistency at the time of collection. Any missing items were immediately verified and completed with the participant whenever possible. As a result, no item-level missing data remained in the final 270 included questionnaires.

### 2.6 Statistical methods

Data were analyzed using SPSS 27.0 and R Studio 4.3.3 software. Categorical data were described as frequencies and percentages. Normality tests were performed on all continuous variables. Data conforming to normal distribution were described as mean ± standard deviation, while non-normally distributed data were presented as median and interquartile range [*M* (*Q_1_*, *Q_3_*)]. Group comparisons were performed using the Mann–Whitney U test or Kruskal–Wallis H test, as appropriate. Spearman correlation analysis was used to assess bivariate associations.

This study aimed to investigate the variables associated with discharge readiness in patients with brucellosis. The total discharge readiness score was defined as the continuous outcome variable. Variables with statistical significance in the above group comparison tests and Spearman’s correlation analysis were selected as candidate predictor variables. The random forest model was constructed using the randomForest package in R. All hyperparameters of the random forest model were adopted as the default settings predefined for regression tasks in this package. The key hyperparameters were specified as follows: the number of decision trees (ntree) = 500; the number of independent variables randomly selected at each node split (mtry) = 3, accounting for one-third of the total 11 included predictors; the minimum sample size of each terminal node (nodesize) = 5. The parameter importance = TRUE was set to generate variable importance metrics. Feature importance was ranked based on the increase in mean squared error (IncMSE) score. Notably, the random forest model adopted in this study was only used for exploratory variable importance ranking, rather than developing or validating a clinical prediction model.

To further identify the core influencing variables of discharge readiness, Lasso penalized regression was performed on the 11 statistically significant variables screened in the preliminary analysis using the glmnet package in R Studio. This approach effectively eliminated redundant variables and resolved multicollinearity among independent variables. The key variables screened by Lasso regression based on L1 regularization constraints were incorporated into the multiple linear regression model to explore their independent effects on discharge readiness. For the subsequent multiple linear regression model, the variable inclusion and exclusion criteria were set at *α_enter_* = 0.05 and *α_exit_* = 0.10, respectively. For model diagnosis, tolerance and variance inflation factor (*VIF*) were calculated to assess multicollinearity, and residual distribution was examined to verify model fitness. All statistical tests were two-tailed, and a *p* < 0.05 was considered statistically significant.

## 3 Results

### 3.1 Questionnaire collection

A total of 275 questionnaires were distributed. Of these, 2 were not returned. Among the 273 returned questionnaires, 1 was excluded because of logical inconsistency and 2 were excluded because of repetitive response patterns. Finally, 270 valid questionnaires were included in the analysis, yielding an effective response rate of 98.2%.

### 3.2 Scores of key assessment scales

The scores of RHDS, K10, MSPSS, and QDTS among patients with brucellosis were as follows: 88.00(73.00, 100.25), 24.00(18.00, 29.00), 58.00(48.75, 66.25), 175.00(150.00, 199.00). Detailed data are presented in Table 1.

**Table 1:** Variables scores among brucellosis patients.

Projects	Number of items	Theoretical score range	Actual score [*M*(*Q_1_*, *Q_3_*)]	Average score per item [*M*(*Q_1_*, *Q_3_*)]
RHDS	12	0 ~ 120	88.00(73.00, 100.25)	7.33(6.08, 8.35)
Personal status	3	0 ~ 30	20.00(16.00, 24.00)	6.67(5.33, 8.00)
Adaptive capacity	5	0 ~ 50	36.00(29.00, 43.00)	7.20(5.80, 8.60)
Anticipated support	4	0 ~ 40	32.00(26.00, 37.00)	8.00(6.50, 9.25)
K10	10	10 ~ 50	24.00(18.00, 29.00)	2.40(1.80, 2.90)
MSPSS	12	12 ~ 84	58.00(48.75, 66.25)	4.83(4.06, 5.52)
QDTS	24	0 ~ 240	175.00(150.00, 199.00)	7.29(6.25, 8.29)

### 3.3 Correlation analysis between variables

Spearman correlation analysis showed that RHDS was negatively correlated with K10 in patients with brucellosis (*r* = −0.249, *p* < 0.001). Conversely, RHDS was positively correlated with both MSPSS and QDTS, with correlation coefficients of *r* = 0.271 and *r* = 0.413, respectively (both *p* < 0.001).

### 3.4 Univariate analysis of discharge readiness

Among the 270 patients, the predominant characteristics were male (75.9%), age 45–59 years (41.5%), educational level at primary school or below (54.4%), and occupation as farmer or herdsman (56.3%). Detailed demographic and clinical characteristics are presented in Table 2. Univariate analysis showed statistically significant differences in RHDS scores among patients with brucellosis across different age groups, educational levels, occupations, household income, marital status, medical expense payment methods, residency, and the presence of symptoms (all *p* < 0.05) as shown in Table 2.

**Table 2:** General information and univariate analysis of readiness for hospital discharge on patients with brucellosis.

Variables	*n*(%)	Score[*M*(*Q*_1_, *Q*_3_)]	*Z*/*H*	*P*
Gender			−1.702	0.089
Male	205(75.9)	88.00(74.00, 102.50)		
Female	65(24.1)	85.00(69.00, 97.00)		
Age (years)			11.661	0.003
18 ~ 44	89(33.0)	96.00(79.50, 106.00)		
45 ~ 59	112(41.5)	86.00(72.00, 98.75)		
≥60	69(25.5)	83.00(72.50, 93.00)		
Educational level			19.574	<0.001
Primary school or below	147(54.4)	84.00(72.00, 93.00)		
Junior middle school	65(24.1)	90.00(72.00, 105.00)		
Senior high school or technical secondary school	31(11.5)	96.00(74.00, 106.00)		
College and above	27(10.0)	99.00(82.00, 114.00)		
Occupation			12.832	0.025
Unemployed	27(10.0)	83.00(75.00, 95.00)		
Organizational/Enterprise Employee	21(7.8)	93.00(82.00, 104.50)		
Laboring	29(10.7)	96.00(71.50, 107.00)		
Farming	152(56.3)	85.50(72.00, 96.00)		
Self-employed	17(6.3)	93.00(71.50, 105.00)		
Other	24(8.9)	103.00(81.00, 114.25)		
Monthly per capita household income (RMB)			13.306	0.004
≤1,000	108(40.0)	83.00(71.25, 95.50)		
1,001 ~ 3,000	102(37.8)	90.00(75.00, 100.00)		
3,001 ~ 5,000	34(12.6)	88.50(75.50, 102.50)		
≥5,001	26(9.6)	103.00(80.25, 115.25)		
Marital status			10.028	0.007
Unmarried	39(14.4)	100.00(81.00, 114.00)		
Married	227(84.1)	87.00(72.00, 97.00)		
Divorced/widowed	4(1.5)	90.50(48.25, 115.50)		
Medical Expense Payment Methods			12.580	0.002
Resident Medical Insurance	213(78.9)	86.00(72.00, 97.00)		
Employee Medical Insurance	46(17.0)	97.50(81.00, 108.50)		
Self-charging	11(4.1)	93.00(73.00, 109.00)		
Residency			−2.456	0.014
Urban	45(16.7)	94.00(83.50, 105.00)		
Rural	225(83.3)	87.00(72.00, 98.00)		
Presence of symptoms			−2.278	0.023
No	5(1.9)	113.00(92.00, 120.00)		
Yes	265(98.1)	88.00(73.00, 100.00)		

### 3.5 Screening of variables related to discharge readiness

#### 3.5.1 Importance ranking via random forest model

**Figure 1:**
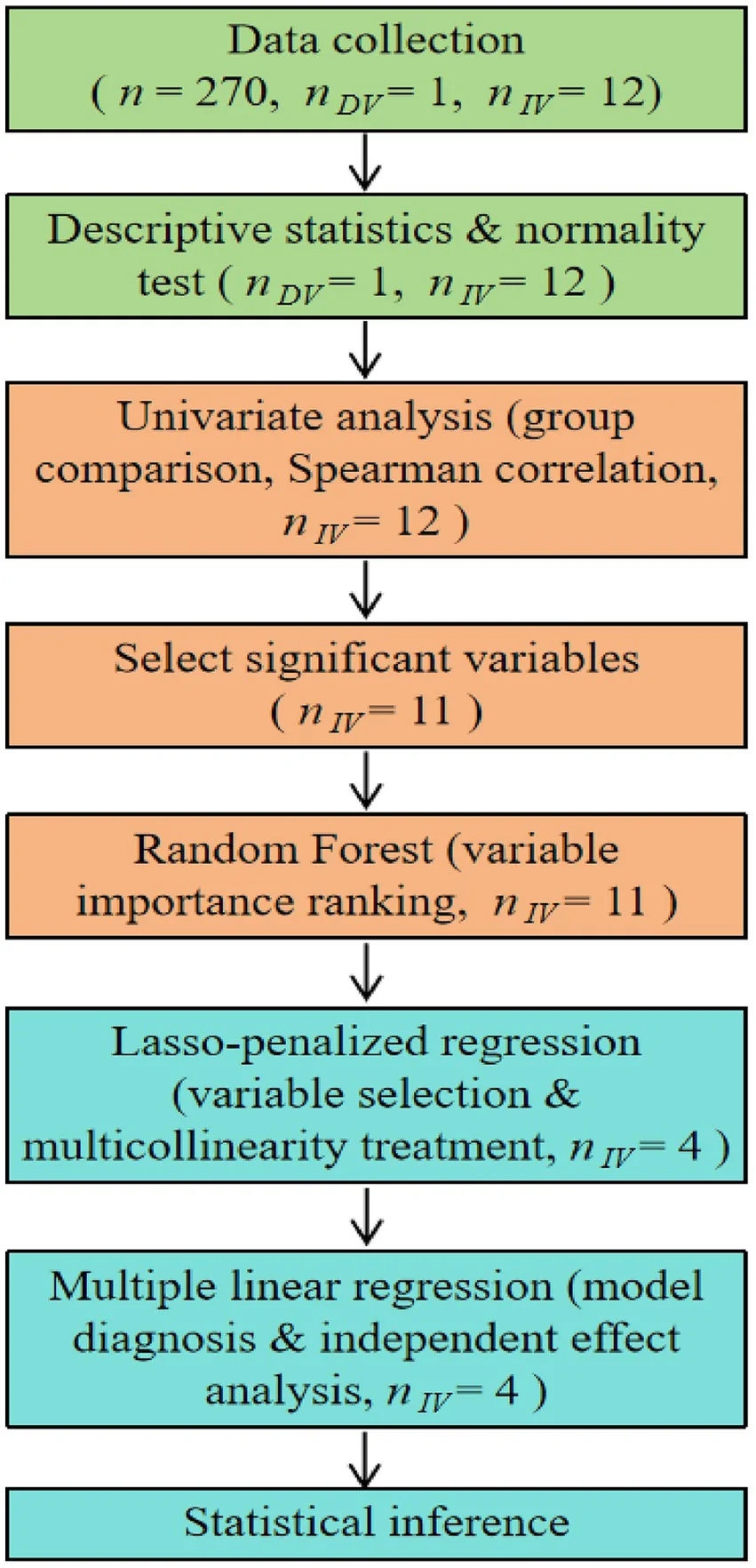
Flow chart of the statistical analysis in this study. *n*: sample size; *nDV*: dependent variable (readiness for hospital discharge of brucellosis patients); *nIV*: independent variable (general demographic and disease-related indicators of brucellosis patients).

**Figure 2:**
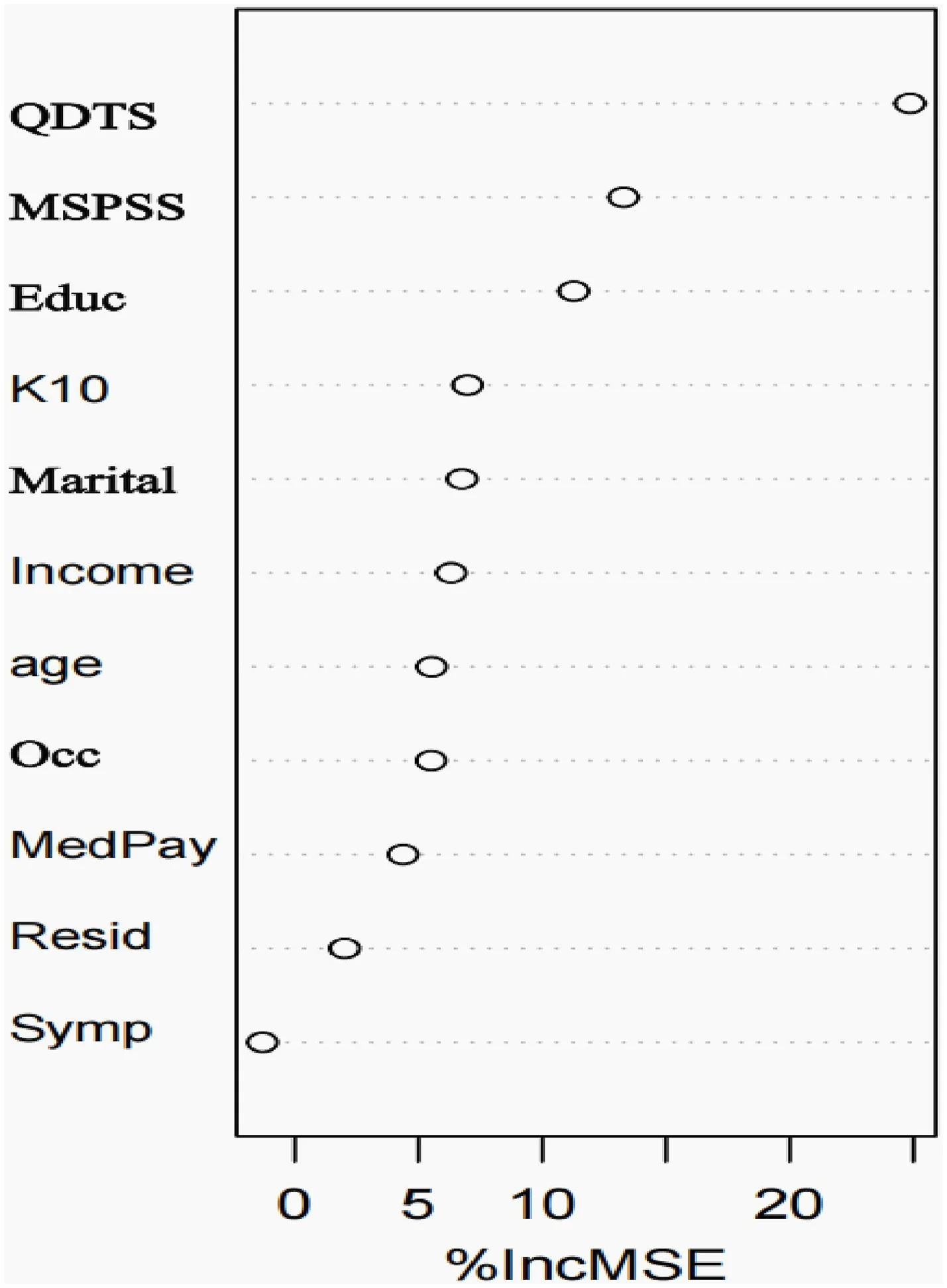
Variable importance ranking based on random forest analysis. Educ, educational level; Marital, marital status; Income, monthly per capita household income (RMB); Occ, occupation; MedPay, medical expense payment method; Resid, residency; Symp, presence of symptoms.

The total RHDS score of patients with brucellosis was set as the dependent variable, and 11 variables with statistically significant differences in univariate analysis and correlation analysis were included as independent variables in the random forest model. The variables were listed as follows from highest to lowest importance: QDTS, MSPSS, educational level, K10, marital status, Monthly per capita household income (RMB), age, occupation, medical expense payment method, residency, and presence of symptoms. Detailed results are shown in Table 3 and Figures 1, 2.

**Table 3:** Importance scores of variables in random forest model.

Related variables	%IncMSE
Age	5.538
Educational level	11.276
Occupation	5.519
Monthly per capita household income (RMB)	6.325
Marital status	6.761
Medical expense payment method	4.381
Residency	2.021
Presence of symptoms	−1.299
K10	6.984
MSPSS	13.292
QDTS	24.864

#### 3.5.2 Variable screening via Lasso regression

Based on the results of variable importance ranking, Lasso analysis was performed on the 11 variables that exhibited statistically significant differences in univariate and correlation analyses using the glmnet package in R Studio. The optimal model fit was achieved at lambda.1se = 4.02848, which corresponding to 4 variables. Therefore, the top 4 most important variables were included in the subsequent multiple linear regression analysis.

### 3.6 Multivariate linear regression analysis of the screened independent variables for discharge readiness in brucellosis patients

Multiple linear regression analysis was performed with the discharge readiness of patients with brucellosis as the dependent variable, and the top four important variables screened by the random forest model and Lasso analysis as the independent variables (*α_entry_* = 0.05, *α_exit_* = 0.10). The coding scheme for independent variables is presented in Table 4. Collinearity diagnostics showed tolerance values ranging from 0.925 to 0.973 and variance inflation factor values ranging from 1.028 to 1.081, indicating no evidence of problematic multicollinearity. Residual inspection suggested an approximately symmetric distribution around zero, supporting the acceptability of the linear regression model for exploratory association analysis. The results demonstrated that educational level, K10, MSPSS and QDTS were independent influencing variables of discharge readiness (*p* < 0.05), which collectively explained 28.4% of the total variance (see Table 5).

**Table 4:** Independent variable values.

Independent variable	Value assignment method
Educational level	Primary school or below = 1, Junior middle school = 2, Senior high school or technical secondary school = 3, College and above = 4
K10	Actual measured value
MSPSS	Actual measured value
QDTS	Actual measured value

**Table 5:** Multivariate regression analysis of factors influencing RHDS in Brucellosis patients (*n* = 270).

Projects	*B*	*SE*	*β*	*t*	*P*
Constant	39.787	8.427	-	4.722	<0.001
Educational level	3.604	1.126	0.167	3.199	0.002
K10	−0.430	0.156	−0.148	−2.757	0.006
MSPSS	0.328	0.090	0.193	3.637	<0.001
QDTS	0.179	0.025	0.378	7.170	<0.001

*F* = 27.712, *P* < 0.001, *R*^2^ = 0.295, Adjusted *R*^2^ = 0.284.

## 4 Discussion

The present study demonstrated that the mean score of discharge readiness among patients with brucellosis was 7.33 (range: 6.08–8.35), corresponding to a moderate level. This result is consistent with those reported by Zhang et al. (28) among patients with acquired immunodeficiency syndrome and Tang et al. (29) among patients with drug-resistant pulmonary tuberculosis. Among all subdimensions, the personal status dimension yielded the lowest score. This pattern may be closely related to the high incidence of musculoskeletal involvement in brucellosis, which has been reported to range from 2 to 77% (8). The main clinical manifestations of musculoskeletal injury include myalgia, arthralgia, and limited mobility, and such symptoms typically require a long recovery period. Restricted by the length of hospital stay, patients often remain symptomatic at discharge, leading to certain deficits in self-care ability. Previous studies have reported that 34.2–44.7% of patients with brucellosis experience difficulties in daily activities or impaired mobility (14), and independent functional capacity has been confirmed as a key variable strongly associated with discharge readiness (30). As one of the five major global zoonotic diseases, brucellosis causes annual economic losses of over 3 billion US dollars worldwide, attributable to patient medical expenditures, labor force loss, and livestock disease prevention and control inputs (2). From the perspective of human-animal cross-transmission, persistent bone and joint dysfunction markedly elevates the risk of reinfection among patients who return to livestock breeding environments, Physical mobility restrictions often result in inadequate protective behaviors during lambing and barn cleaning. Accordingly, combined with relevant research evidence and clinical practical demands, early functional rehabilitation intervention is recommended alongside symptomatic pharmacological treatment for brucellosis patients. This integrated strategy can effectively improve patients’ physical and mental status, and enhance their capability and confidence in returning to family life, daily routines, and livestock production activities.

The results of this study revealed a positive correlation between discharge teaching quality and discharge readiness in patients with brucellosis; that is, higher discharge teaching quality was associated with better discharge readiness, which is consistent with previous findings (15). Existing evidence indicates that discharge education is associated with patients’ mastery of disease-related knowledge, and standardized discharge guidance can supplement patients’ disease information and reduce insufficient discharge readiness caused by inadequate health literacy (31). Given the low level of disease awareness among patients with brucellosis (32) and the favorable environment for health education during hospitalization, Based on the correlations identified in this study and the practical demands of clinical care, it is recommended that clinical nurses adopt patient-centered care frameworks guided by standardized nursing theories and health education models. Clinicians should integrate key source-based prevention and control strategies—including livestock immunization, food safety, breeding environment disinfection, and occupational protection protocols—into a standardized health education system (2). Continuous optimization of health education modalities can compensate for the limitations of traditional health education, which merely focuses on clinical diagnosis and treatment for infected individuals, and ultimately improve patients’ comprehensive discharge preparedness.

This study identified a significant positive correlation between perceived social support and discharge readiness in patients with brucellosis, indicating that higher perceived social support was associated with better discharge readiness. This result is consistent with that of Lin et al. (15) among patients with stoma construction after colorectal cancer surgery. Most patients with brucellosis are farmers, herdsmen, and young adult males (3). Following disease onset, they are often unable to continue breeding work and may need to sell cattle, sheep, and other assets to afford treatment. Under the dual pressure of poor health and heavy economic burden (33), patients have an urgent need for diverse supportive resources to facilitate treatment and recovery. After discharge, due to limited self-care capacity and functional mobility, patients rely heavily on family members for long-term rehabilitation. Meticulous daily care, strong medical support, and emotional comfort from family members are associated not only with favorable family functioning but also with improved disease coping ability. A study by Wang et al. (34) among patients with pulmonary tuberculosis showed that patients with dedicated caregivers after discharge exhibited relatively higher discharge readiness. Constrained by limited community medical resources, post-discharge treatment and follow-up of patients with brucellosis still require guidance from specialized professionals. Professional medical support after discharge has also been recognized as an essential component for improving discharge readiness (35). Therefore, the establishment of a multidimensional and collaborative support network integrating home care, community follow-up, livestock technical guidance, and specialized continuous treatment serves as a critical measure to facilitate patients’ early recovery of labor capacity and enable them to resume family and livestock production-related social responsibilities.

The present study found a positive correlation between educational level and discharge readiness in patients with brucellosis, such that patients with higher educational attainment presented higher discharge readiness. This finding is consistent with that reported by Li et al. (36) among patients undergoing total knee arthroplasty. A possible explanation is that patients with higher educational levels possess stronger information comprehension ability and wider access to health knowledge, and sufficient information and knowledge have been identified as core components of discharge readiness (15). As an uncontrollable objective variable, educational level suggests that clinical nurses should include educational background in nursing assessment when providing discharge readiness education for patients with brucellosis, and adopt diversified health education methods to meet the personalized needs of patients with different educational levels. Particular attention should be paid to patients with low educational attainment, for whom teach-back methods, videos, and pictorial materials may be used to gradually promote knowledge-attitude-practice changes and genuinely improve discharge readiness.

This study demonstrated a negative correlation between psychological distress and discharge readiness in patients with brucellosis, meaning that more severe psychological distress was associated with poorer discharge readiness. This result is consistent with that of Bai et al. (14) among patients with chronic kidney disease.

This phenomenon may be attributed to multiple adverse predicaments among patients, including prognosis-related concerns, inadequate family care, loss of livestock income, and heavy household economic burdens. Brucellosis is characterized by diverse clinical presentations, a protracted disease course, and high recurrence rates (34). From the One Health perspective focusing on human-animal coexistence, patients endure dual psychological stress: anxiety caused by physical symptoms and economic pressure stemming from livestock industry losses. As livestock constitute the primary household means of production, brucellosis-induced animal abortion and subsequent suspension of breeding income can continuously aggravate patients’ depressive emotions and catastrophic cognition. This intensifies illness uncertainty, impairs patients’ ability to comprehend and absorb health education and discharge guidance delivered by medical staff, and ultimately diminishes their discharge readiness.

According to the scar theory, anxiety, depression, and accompanying interpersonal deficits contribute to declines in cognitive function and daily living ability, and this scar-like relationship may be exacerbated by stress-related biomarkers such as inflammatory factors (37). While providing symptomatic treatment for brucellosis patients, clinicians are recommended to collaborate with township animal husbandry staff to conduct synchronous health education regarding livestock immunization and economic compensation policies for brucellosis outbreaks. This targeted intervention can effectively relieve patients’ anxiety related to breeding economic losses, improve their psychological status, and ultimately elevate their discharge readiness.

### 4.1 Limitations

This study has several limitations. First, as a single-center cross-sectional study, it cannot establish temporal sequence or causal relationships between variables and discharge readiness, and the external generalizability of the findings is limited. Second, the sample size meets the requirement of exploratory correlation analysis, but it is insufficient for constructing and verifying a robust predictive model.

Third, pre-selecting predictors according to univariate statistical significance prior to LASSO regression can introduce variable selection bias, which may inadvertently exclude variables with important clinical value despite lacking univariate statistical significance. The final multivariate regression model accounted for only 28.4% of the total variance, implying that a large number of key determinants associated with discharge readiness were not measured in this study. We recommend that future investigations incorporate additional clinically relevant indicators, including disease severity, treatment duration, physical functional status, health literacy, and objective clinical endpoints, to strengthen the explanatory power of the regression model.

Fifth, random forest was only deployed as an exploratory approach to rank variable importance, rather than a validated predictive machine learning model. We did not partition the dataset into training and validation subsets, nor did we conduct internal/external validation or calibration for this algorithm. Accordingly, we make no claim that the combined random forest-LASSO analytical strategy outperforms conventional multivariate regression models in terms of analytical performance, and random forest outputs were solely used for descriptive variable screening. Future prospective longitudinal cohort studies are needed to follow the full disease trajectory of patients with brucellosis throughout hospitalization, recovery, and the return to livestock breeding activities. A broader set of One Health indicators involving human, animal and environmental dimensions should be incorporated to further verify variable correlations and explore potential causal associations based on enlarged sample sizes.

## 5 Conclusion

Patients with brucellosis had a moderate level of discharge readiness. Better discharge teaching quality, greater perceived social support, higher educational level, and lower psychological distress were associated with higher discharge readiness. This finding not only helps clinicians quickly and accurately identify the key variables most closely associated with discharge readiness in patients with brucellosis, but also provides a scientific evidence base for subsequent high-efficiency, individualized intervention studies aimed at improving discharge readiness in these patients.

## Data Availability

The raw data supporting the conclusions of this article will be made available by the authors, without undue reservation.
